# Animal model of intrahepatic metastasis of hepatocellular carcinoma: establishment and characteristic

**DOI:** 10.1038/s41598-020-72110-7

**Published:** 2020-09-16

**Authors:** Xuemei Li, Jike Hu, Baohong Gu, Maswikiti Ewetse Paul, Bofang Wang, Yang Yu, Zedong Feng, Yanling Ma, Xueyan Wang, Hao Chen

**Affiliations:** 1 The Second Clinical Medical College of Lanzhou University, Lanzhou, 730000 China; 2 The Department of Tumor Surgery, Lanzhou University Second Hospital, Lanzhou, 730000 China; 3 The Key Laboratory of the Digestive System Tumors of Gansu Province, Lanzhou University Second Hospital, Lanzhou, 730000 China; 4 The Department of Pediatric Surgery, Lanzhou University Second Hospital, Lanzhou, 730000 China

**Keywords:** Cancer models, Metastasis

## Abstract

One of the most important and striking characteristics of hepatocellular carcinoma (HCC) with intrahepatic metastasis, is that it results in extremely poor prognosis. Animal models have become a fundamental and very useful in research for disease study. However, some limitation has arisen from these model systems. We have therefore established a model of HCC with intrahepatic metastasis and noticed some differential appearances in different HCC cell lines. Luciferase-transfected HCC cell lines MHCC97-H and PLC/PRF/5 were inoculated into SCID mice via spleen. Observation the intrahepatic metastasis by bioluminescence imaging in vivo and comparing of the differential formation of metastatic lesions between different HCC cell lines by incorporating physical anatomy was done. Animal models for HCC intrahepatic metastasis were well established. However, there were some clearly noticed differences between MHCC97-H and PLC/PRF/5 cell lines. The group of MHCC97-H cell line readily metastasis in the liver, whereas group PLC/PRF/5 cell line developed extensive intrahepatic metastasis and formed large tumor in situ in the spleen. MHCC97-H and PLC/PRF/5 cell lines can be used to successfully establish a model of HCC intrahepatic metastasis with distinctive characteristics, which provides an important direction for the study of the mechanism of HCC intrahepatic metastasis, and may hopefully provide a basis for clinical treatment.

## Introduction

Hepatocellular carcinoma is the most common disease with malignant tumors of the liver and the third resulting in cancer mortalities in the world^1^. Surgery has become the most effective therapeutic strategic method in managing HCC patients, but the recurrence rate is still as high as 80%, and most of this recurrence occurs within 1–2 years post operatively^2^. The most common form of HCC recurrence comes as a result of intrahepatic metastasis. The reresection rate after recurrence is up to 10.4–31%, but the 3-year survival rate after reresection is only 52.6%^3^. Therefore, recurrence and metastasis are the main factors affecting the survival rate and contributing to prognosis in HCC patients. The exact mechanism under HCC recurrence and intrahepatic metastasis need to be explored by the utilization of animal models, however, the appropriate animal model of intrahepatic metastasis is not well established and verified.

There are several HCC intrahepatic metastasis animal models. First and foremost, directly implanted cell lines^4^ or tumor tissues^5^ into the liver and then form metastasis, which is most similar to the in-vivo condition of HCC intrahepatic metastasis. However, the application of this animal model is limited due to the destruction of the original morphological structure of the liver and the difficulty in arresting and controlling bleeding. There is also a long waiting time for formation and progression of intrahepatic metastasis. Secondly, an injection administration of HCC cells via portal vein is also an ideal method for intrahepatic metastasis. Nevertheless, it is difficult to perform an operation and arrest bleeding and this gives rise to a lower postoperative survival rate^6^. Finally, the injection administration of HCC cell into spleen is widely used to establish intrahepatic metastasis model due to its simple operation and high rate of tumor formation. It was found that the hepatoma cell suspension being injected into the spleen not only formed the tumor in situ, but also the definite metastasis in the liver^7,8^.

HCC cell line PLC/PRF/5 which was established by Alexander et al.^9^, produces HBsAg. PLC/PRF/5 is moderately invasive and be widely used in basic research. Studies have found that HBV and AFB1 (Aflatoxin B1) induces and gives rise to the development of HCC. Human HCC cell line MHCC97-H appears as polygonal epithelial cells with metastatic potential, which can secrete AFP. It has been found that these cell lines after intrahepatic inoculation can lead to liver, diaphragm and abdominal wall invasion^10,11^. In this study, we used PLC/PRF/5 and MHCC97-H HCC cell lines to establish HCC intrahepatic metastasis models and observed the differences between both cell lines. Our study paved a way for the fundamental research of HCC intrahepatic metastasis.

## Materials and methods

### Mice

Severe combined immunodeficiency (SCID) CB-17 mice purchased from Weitonglihua (Beijing, China) were used in our study. All animals were housed in a SPF condition in our lab. Mice were operated at 6–8 weeks and their body weight ranged from 18 to 22 g. All animal experimental procedures were in accordance with ARRIVE guidelines.

### Cell culture

HCC cell lines MHCC97-H and PLC/PRF/5 were cultured in DMEM (Hyclone, USA) supplemented with 10% FBS (Invitrogen) and 1% penicillin (Invitrogen) in an incubator under a 5% carbon dioxide atmosphere at 37 °C in a relative humidity of 95%.

### Virus transfection

Luciferase negative control virus purchased from Genechem Chemtech (Shanghai, China) was utilized in our study, and the sequence was 5′-TTCTCCGAACGTGTCACGT-3′. Virus transfection were operated as follows. PLC/PRF/5 and MHCC97-H cells were cultured in 6-well plates at a concentration of 2 × 10^5^ per plate and prepared of transfection. Multiplicity of infection (MOI) of PLC/PRF and MHCC97-H are 10 and 30 respectively. Then viruses were transfected into both cells and using polybrene (10 ug/ml) to enhance transfection efficiency. After 12 h of incubation, the medium was replaced with a fresh DMEM complete medium. After incubation for 48–72 h, cells were observed by an in vivo imaging system by adding d-Luciferin, Sodium Salt to evaluate the efficiency of transfection.

### Animal operations

All animal studies complied by incorporating National Institutes of Health guidelines for the care and use of Laboratory animals were approved by the Lanzhou University Second Hospital Institutional Animal Care and Use committee (D2017-052). Mice were grouped into PLC/PRF/5 group (n = 3) and MHCC97-H group (n = 6) respectively. All cells transfected with virus were used and injected into spleen of mice using an insulin needle. The cellular concentration was 1 × 10^6^/100 ul complete medium, which were completely injected into spleen within a minute. Postoperatively all mice were placed on a warm thermal generator until they were fully restored to a normal condition. Mice were finally kept under close observation and monitoring under SPF conditions.

### Bioluminescence assay

In vivo Bioluminescence assays were performed on days 4 and 14 postoperatively. All mice were intraperitoneally injected with 150ul 15 mg/ml d-Luciferin, Sodium Salt. After 10 min, mice were anesthetized with isoflurane gas and placed into the imaging chamber and X-ray imaging was performed. Post X-ray shooting bioluminescence imaging was done.

### Statistical analysis

For the statistical analysis SPSS22.0 software was used. Numerical data was analyzed using the Student’s t test. A difference was considered significant when the *P* value was < 0.05.

## Results

### Establishment of HCC cell lines with a stable expression of luciferase

**Figure 1:**
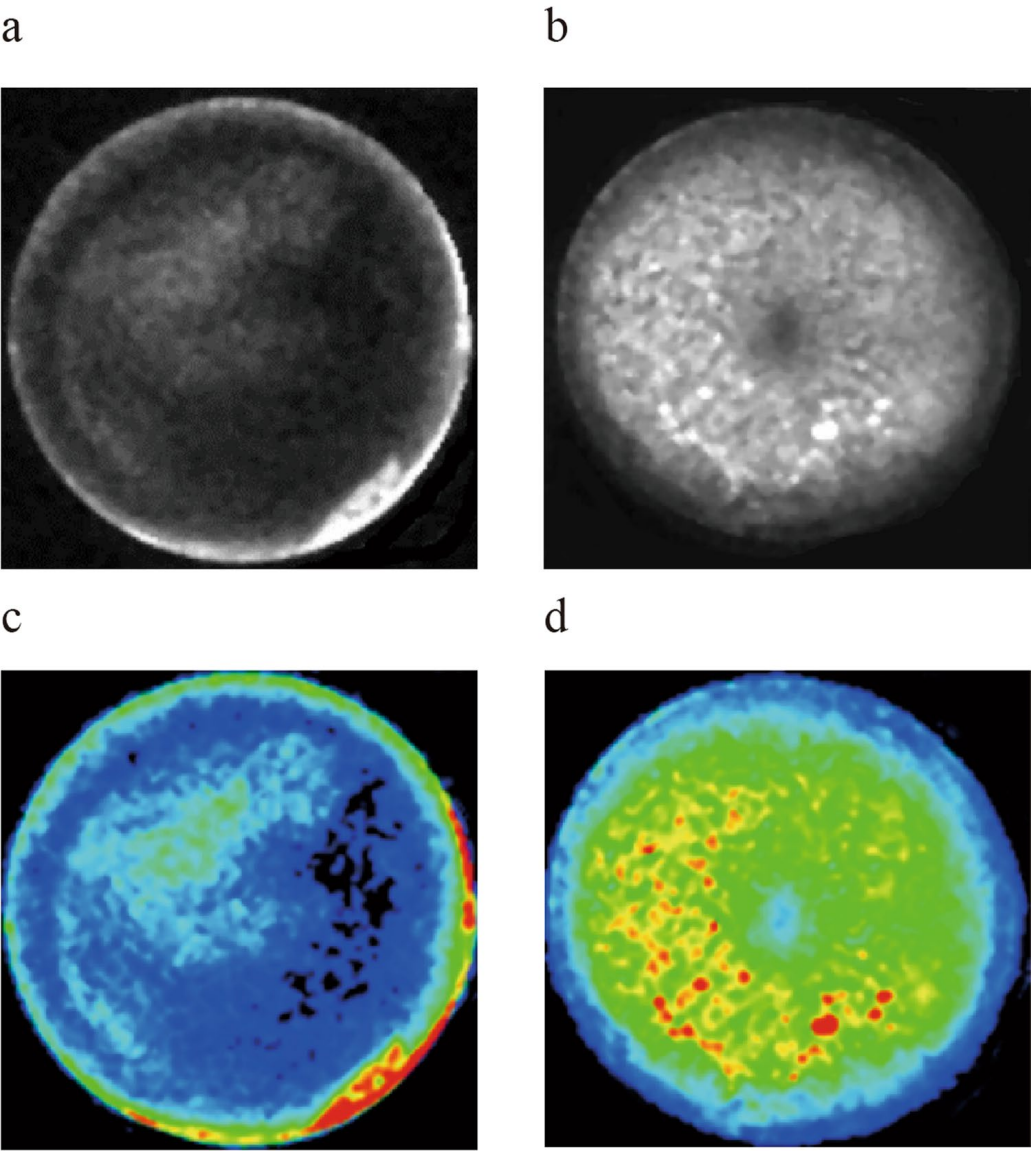
Bioluminescence and luciferase assay of HCC cell lines. (**a**–**c**) PLC/PRF/5 cell line in the blank and fluorescent vision. (**b**–**d)** MHCC97-H cell line in the blank and fluorescent vision.

After transfecting with luciferase virus, the efficiency of transfection was evaluated by the utilization of bioluminescence and luciferase assays. Results showed that PLC/PRF/5 (Fig. 1c) and MHCC97-H (Fig. 1d) cells were transfected successfully, the efficiency of transfection was up to 80%.

### Success in the establishment of intrahepatic metastasis model

**Figure 2:**
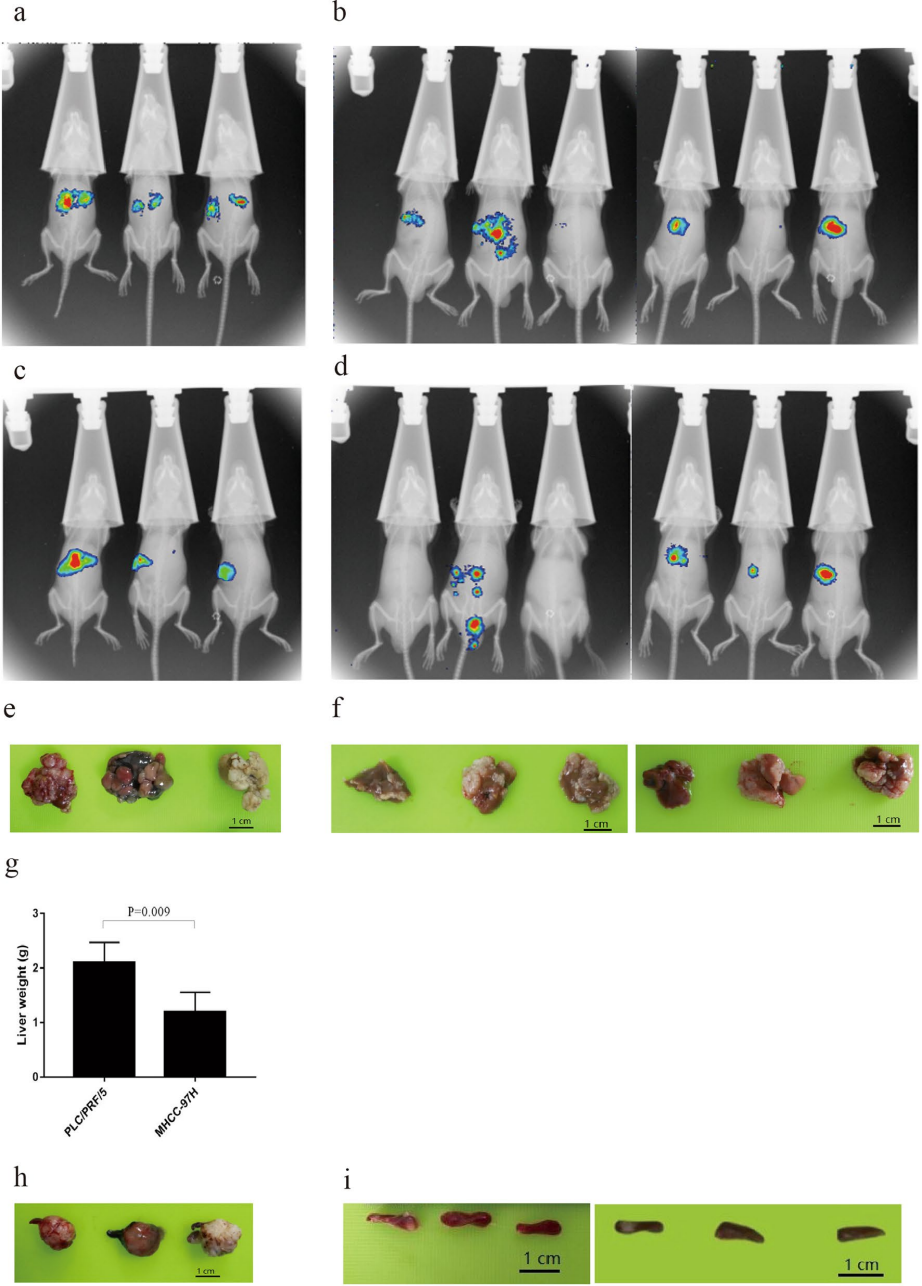
In vivo Bioluminescence assay and physical anatomy after operation. (**a**,**b**) PLC/PRF/5 and MHCC97-H groups on day 4 post-operation respectively. (**c**,**d**) PLC/PRF/5 and MHCC97-H groups on day 14 post-operation respectively. (**e**) Intrahepatic metastasis lesions of PLC/PRF/5 group. (**f**) Intrahepatic metastasis lesions of MHCC97-H group. (**g**) Comparison of liver weight between two groups. (**h**) Spleen orthotopic tumor of PLC/PRF/5 group. (**i**) Spleen orthotopic tumor of MHCC97-H group.

In vivo Bioluminescence assays were first performed on day 4 post-operation, which indicated that both groups PLC/PRF/5 (Fig. 2a) and MHCC97-H with bioluminescence (Fig. 2b) were can be found in liver in vivo. This proved that our intrahepatic metastasis animal models were successfully established. In vivo Bioluminescence assays were also performed on day fourteen post-operation, which illustrated that both groups were observed and had multiple intrahepatic metastasis (Fig. 2c,d).

### The difference in tumor formation between PLC/PRF/5 and MHCC97-H cell lines

Next, we euthanized the mice at twenty-five (PLC/PRF/5) (Fig. 2e) and at day 35 (MHCC97-H) (Fig. 2f) post-operation respectively and results showed that the liver of the former is significantly heavier than that of the latter (*P* < 0.05) (Fig. 2g).

Most importantly, we observed that there was a vast difference of gross specimen of the spleen between the two groups. As it could be seen, huge in situ tumors were formed in the spleen of PLC/PRF/5 group (Fig. 2h), but there was almost no tumor formation in the spleen of MHCC97-H group (Fig. 2i).

## Discussion

Intrahepatic multiple metastasis and recurrence are typical features and signs of HCC, which always lead to very poor disease prognosis in these patients. However, the mechanism of this phenomenon is still very unclear. The high incidence and the unclarity of HCC intrahepatic metastatic disease process and manifestation has made researchers to come up with an appropriate animal model to try and understand the disease process.

First of all, we established HCC intrahepatic metastasis model by injecting suspended HCC cell lines into spleen. This method is not only simple to operate, but has a high rate of tumor formation. However, it leads simply to, effective bleeding and difficulties in coagulation processes since the spleen is a very fragile organ. Furthermore, we transfected control virus with luciferase into HCC cell lines, which was convenient to evaluate the distribution and growth of tumors in vivo in real time.

We selected two HCC cell lines with different degrees to explore their potential for intrahepatic metastasis respectively. In comparison, MHCC97-H is a highly metastasis potential cell line, while PLC/PRF/5 is not. After operation we performed an in vivo Bioluminescence assay on days 4 and 14 post-operation, which revealed that both of cell lines could form tumors in vivo and multiple intrahepatic metastasis lesions were also clearly observed. This meant that both cell lines had a strong tumor-forming potential and could be used to establish animal models of HCC.

Next, we compared the difference in tumor formation between the two cell lines. First of all, it could be clearly noticed that MHCC97-H group had a longer survival time than PLC/PRF/5 group at the 25 and 35 day respectively. So, we dissected the mice at different time and found out that the livers of PLC/PRF/5 group were significantly heavier than those in the MHCC97-H group. Most importantly, huge tumors in situ were formed in the spleen of PLC/PRF/5 group, while there was almost no tumor formation in the spleen of MHCC97-H group. A study comparing the growth capacity of transplantable tumors in several organs found that two of the hepatomas grew much more readily and vividly in the spleen than in any other organs elsewhere. They suggested that the difference in their capacity to metastasize is related to the differences in cell adhesiveness. The spleen seems to be structurally well adapted to permit the release of non-adhesive cells into the blood stream^7,8^. Therefore, we speculate that PLC/PRF/5 cells are more likely to adhere and have adhesion properties than MHCC97-H cells. However, it is not clear at the present moment what really contributes to PLC/PRF/5 cells to have a stronger ability of adhesion and further research is needed to elucidate this phenomenon.

In summary, the SCID mice model established here have similarities with HCC intrahepatic metastasis patients. HCC cell lines in both MHCC97-H and PLC/PRF/5 are tumorigenic and have a great potential for intrahepatic metastasis. In addition, the two cell lines are different in their spleen appearance, while the specific mechanism is not explicit and needs further investigations and study. Overall, it is definite that our model system will be a useful tool for the basic research of intrahepatic metastasis of HCC and can well provide researchers with the broad base of cell lines selection.

## Supplementary information

Supplementary file1
