# Supplementary material for: Animal model of intrahepatic metastasis of hepatocellular carcinoma: establishment and characteristic
Source: Sci Rep. 2020 Sep 16;10:15199. doi: 10.1038/s41598-020-72110-7 (PMC7494875; doi:10.1038/s41598-020-72110-7)
Supplement: Supplementary file 1 — Supplementary file1 [file 41598_2020_72110_MOESM1_ESM.pdf]

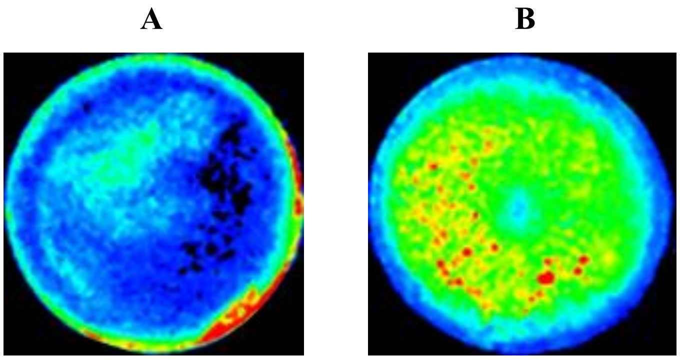

**Figure 1**. Aioluminescence and luciferase assay of HCC cell lines. (**A)** PLC/PRF/5 cell line. (**B)** MHCC97-H cell line.

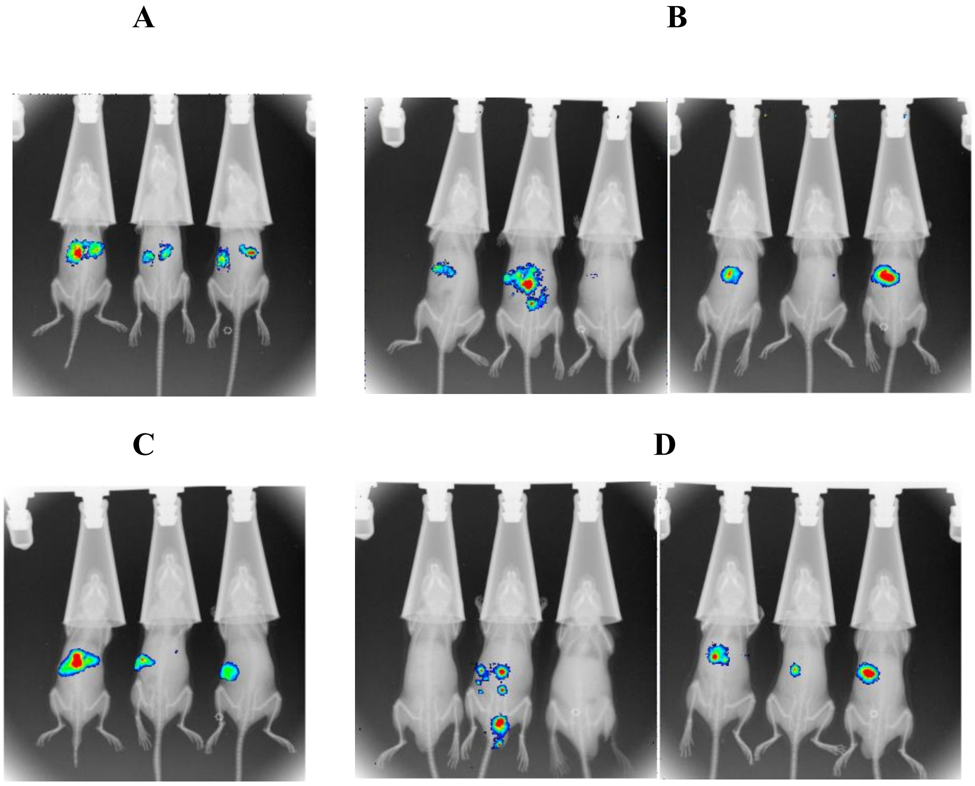

**Figure 2**. In vivo Bioluminescence assay after operation. (**A-B**) PLC/PRF/5 and MHCC97-H groups on the 4day post-operation respectively. (**C-D**) PLC/PRF/5 and MHCC97-H groups on the 14day post-operation respectively.

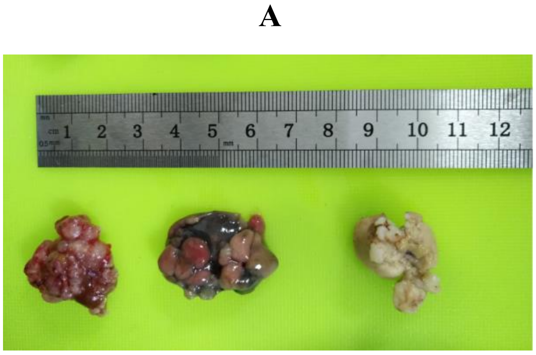

**B**

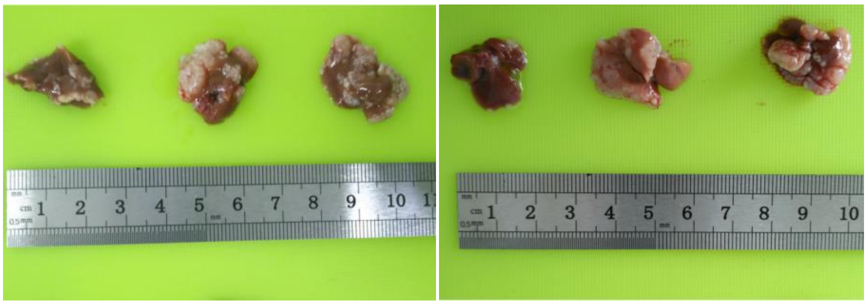

**C**

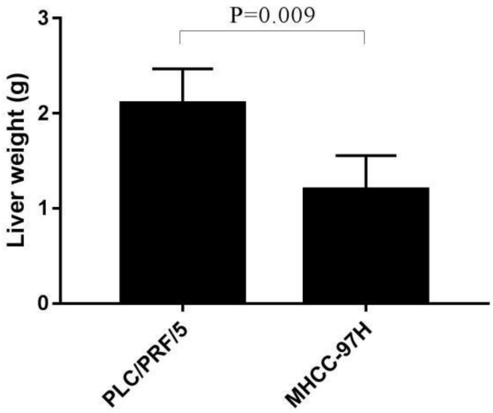


**Figure 3**. Intrahepatic metastasis of Hepatocellular carcinoma. (**A**) Intrahepatic metastasis lesions of PLC/PRF/5 group. (**B**) Intrahepatic metastasis lesions of MHCC97-H group. (**C**) Comparsion of liver weight between two groups.

**A**

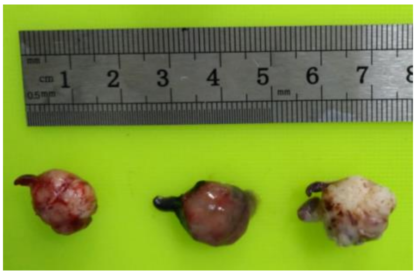

**B**

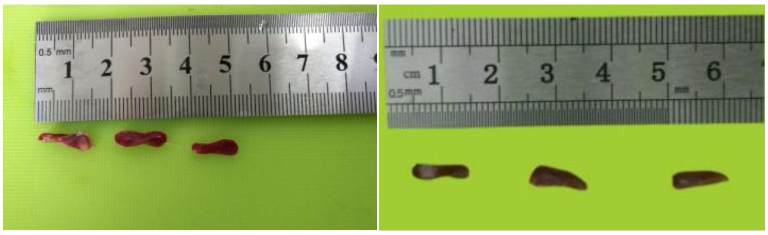

**Figure 4**. Carcinoma in situ of spleen. (**A**) Spleen orthotopic tumor of PLC/PRF/5 group. (**B**)Sspleen orthotopic tumor of MHCC97-H group.
